# Continuation With Statin Therapy and the Risk of Primary Cancer: A Population-Based Study

**DOI:** 10.5888/pcd9.120005

**Published:** 2012-08-09

**Authors:** Miriam Lutski, Varda Shalev, Avi Porath, Gabriel Chodick

**Affiliations:** Author Affiliations: Miriam Lutski, Sackler Faculty of Medicine, Tel Aviv University, Israel; Varda Shalev, Sackler Faculty of Medicine, Tel Aviv University, Israel, and Maccabi Healthcare Services, Tel Aviv, Israel; Avi Porath, Maccabi Healthcare Services, Tel Aviv, Israel.

## Abstract

**Introduction:**

Studies have suggested that statins may inhibit tumor cell growth and possibly prevent carcinogenesis. The objective of this study was to investigate the association between persistent statin use and the risk of primary cancer in adults.

**Methods:**

This retrospective study was conducted by using the computerized data sets of a large health maintenance organization (HMO) in Israel. The study population was 202,648 enrollees aged 21 or older who purchased at least 1 pack of statin medication from 1998 to 2006. The follow-up period was from the date of first statin dispensation (index date) to the date of first cancer diagnosis, death, leaving the HMO, or September 1, 2007, whichever occurred first. Persistence was measured by calculating the mean proportion of follow-up days covered (PDC) with statins by dividing the quantity of statin dispensed by the total follow-up time.

**Results:**

During the study period, 8,662 incident cancers were reported. In a multivariable model, the highest cancer risk was calculated among nonpersistent statin users. A strong negative association between persistence with statin therapy and cancer risk was calculated for hematopoietic malignancies, where patients covered with statins in 86% or more of the follow-up time had a 31% (95% confidence interval, 0.55-0.88) lower risk than patients in the lowest persistence level (≤12%) .

**Conclusion:**

Our study demonstrated that persistent use of statins is associated with a lower overall cancer risk and particularly the risk of incident hematopoietic malignancies. In light of widespread statin consumption and increases in cancer incidence, the association between statins and cancer incidence may be relevant for cancer prevention.

## MEDSCAPE CME

Medscape, LLC is pleased to provide online continuing medical education (CME) for this journal article, allowing clinicians the opportunity to earn CME credit.

This activity has been planned and implemented in accordance with the Essential Areas and policies of the Accreditation Council for Continuing Medical Education through the joint sponsorship of Medscape, LLC and Preventing Chronic Disease. Medscape, LLC is accredited by the ACCME to provide continuing medical education for physicians. 

Medscape, LLC designates this Journal-based CME activity for a maximum of 1 **AMA PRA Category 1 Credit(s)™**. Physicians should claim only the credit commensurate with the extent of their participation in the activity.

All other clinicians completing this activity will be issued a certificate of participation. To participate in this journal CME activity: (1) review the learning objectives and author disclosures; (2) study the education content; (3) take the post-test with a 70% minimum passing score and complete the evaluation at www.medscape.org/journal/pcd (4) view/print certificate.

**Release date: August 08, 2012; Expiration date: August 08, 2013**

### Learning Objectives

Upon completion of this activity, participants will be able to:

- Assess the pleiotropic effects of statins
- Distinguish the effect of statin therapy on the risk of incident cancer in the current study
- Identify the type of cancer most inhibited by statins in the current study
- Evaluate variables which might affect the efficacy of statins in preventing cancer

**CME EDITOR**

Teresa Ramsey, Editor; Camille Martin, Editor, *Preventing Chronic Disease*. Disclosures: Teresa Ramsey and Camille Martin disclosed no relevant financial relationships.

**CME AUTHOR**

Charles P. Vega, MD, Health Sciences Clinical Professor; Residency Director, Department of Family Medicine, University of California, Irvine. Disclosure: Charles P. Vega, MD, has disclosed no relevant financial relationships.

**AUTHORS AND CREDENTIALS**

Disclosures: Miriam Lutski, MSc; Varda Shalev, MD; Avi Porath, MD; Gabriel Chodick, PhD have disclosed no relevant financial relationships.

Affiliations: Miriam Lutski, MSc, Sackler Faculty of Medicine, Tel Aviv University, Israel; Varda Shalev, MD, Sackler Faculty of Medicine, Tel Aviv University, Israel, and Maccabi Healthcare Services, Tel Aviv, Israel; Avi Porath, MD, Gabriel Chodick, PhD, Maccabi Healthcare Services, Tel Aviv, Israel

## Introduction

Cancer is the second most common cause of death in the United States, exceeded only by heart diseases (1). Annual deaths from cancer are projected to continue rising and are estimated to be 17 million worldwide in 2030 (2). Cancer is the leading cause of death in Israel (approximately 25% of all-cause mortality) and is a major cause of morbidity in the population (3).

3-Hydroxy-3-methylglutaryl-coenzyme A (HMG-CoA) reductase inhibitors (statins) effectively reduce cholesterol levels and decrease the incidence of cardiovascular and cerebrovascular events (4). Large randomized controlled trials (RCTs) that examined the safety and effectiveness of statins in preventing cardiovascular diseases indicated that statins were not associated with increased cancer risk (5). However, these RCTs were limited to short-term follow-up, the duration of which was insufficient to adequately evaluate carcinogenesis risk (6).

After statins were introduced into clinical practice, they were shown to have effects other than lipid lowering, referred to as pleiotropic effects (7). More than 15 years ago, cholesterol decrement was first shown to inhibit tumor cell growth, metastasis of tumor cells, and induction of apoptosis (7). Inhibition of HMG-CoA reductase by statin in effect prevents the synthesis of mevalonic acid, a precursor of nonsteroidal isoprenoids, lipid attachment molecules for small G proteins, such as Ras, Rho, and Rac. Thus, statins may inhibit the synthesis of isoprenoids and thereby suppress the activation of small G proteins (7).

Statins have been associated with a significantly lower risk of breast (8), colorectal (9), and lymph cancers (10-12) in several observational studies (10,13). Most previous observational studies have been limited by a small number of cases, short follow-up period, unverified self-reports on use and consistency of statins therapy, and no assessment of statins efficacy.

Our previous study (14) demonstrated a strong negative association between persistent use of statins and all-cause mortality reduction among patients with and without a history of coronary heart disease (CHD). The observed reduced mortality in statin users cannot be explained by lower incidence of CHD death alone. The objective of this study was to investigate the association between persistent use of statins and the risk of overall and site-specific cancer in adults, to assess dose-response relationship, and to examine the effects of varying types of statins. We focused on breast and genitourinary, colorectal, lung and bronchus, prostate, leukemia, hematopoietic, and lymphoma malignancies.

## Methods

We conducted this study among members of Maccabi Healthcare Services (MHS), established in 1941. MHS has become Israel’s second-largest health maintenance organization, with a membership of 2 million countrywide. All data for this analysis were obtained from MHS automated databases.

The cohort of statin users has been described previously (14,15). Briefly, the study was conducted with a follow-up spanning the period between the date of first dispensed statin to the date of cancer diagnosis, death, leaving MHS, or September 1, 2007, whichever occurred first. New users of statins were identified among all MHS enrollees aged 21 or older on January 1, 1998, who had at least 1 dispensed prescription of statin medications from January 1, 1998, to September 1, 2006; the date of first dispensation was classified as the index date. We included only patients who had no record of purchasing statin medication before the index date to allow for evaluation of new users. A total of 227,131 new users of statin medications were eligible for analysis. We excluded all patients who were diagnosed with cancer before the index date (n = 12,499). To avoid incidence-prevalence bias, we excluded cases diagnosed with cancer within 1 year from index date, and we excluded all patients with a minimal exposure period of statins under 1 year, the period required for statin medication to have any effect on the development of cancer (n = 11,984). After applying the inclusion and exclusion criteria, 202,648 (89%) patients were eligible for analysis.

Data on cancer occurrence during the study follow-up period were obtained from the Israel National Cancer Register (ICR). Established in 1960, the ICR collects information on diagnosed cancer cases from all medical institutions in the country with a completeness of above 93.5% for solid tumors and approximately 90% for nonsolid tumors (3). We classified all cancer cases according to the 3rd edition of the International Classification of Diseases for Oncology (ICD-O). All cases are based on histological reports, hospital discharge forms, oncology reports, and death certificates. Approximately 92% of registered cases had a valid histology or cytology report. The study population and the ICR were cross-linked by the members’ unique identifying number, given to all newborns or immigrants to Israel; name; sex; and date of birth.

Following previous categorization of statin therapy (16), we categorized initial statin therapy into 3 efficacy levels that were created on the basis of expected amounts of low-density lipoprotein cholesterol (LDL-C) reduction from baseline: low efficacy (daily dose of simvastatin, 10 mg or less; pravastatin, 10 mg; fluvastatin, 40 mg or less; lovastatin, 20 mg or less; or cerivastatin, 0.2 mg), moderate efficacy (daily dose of simvastatin, 20 mg; pravastatin, 20 mg or 40 mg; fluvastatin, 80 mg; lovastatin, 40 mg; atorvastatin, 10 mg; rosuvastatin, 10 mg or less; or cerivastatin, 0.3 or 0.4 mg), or high efficacy (daily dose of simvastatin, 40 mg or 80 mg; pravastatin, 80 mg; lovastatin, 80 mg; atorvastatin, 20 mg or more; rosuvastatin, 20 mg or more; or cerivastatin, 0.8 mg).

Continuation with statin therapy was individually assessed by calculating the mean proportion of follow-up days covered (PDC) with statins by dividing the quantity of statin packs dispensed by the total follow-up days. PDC was categorized into quintiles (≤12%, 13%–39%, 40%–66, 67%–85%, and ≥86%).

Demographic variables at index date included baseline values of age, sex, marital status, place of residency, years of stay in Israel (for new immigrants) and religiosity (categorized into ultra-orthodox Jewish, other Jewish, and non-Jewish). These categories were determined on the basis of self-reported data obtained by MHS for marketing purposes. Socioeconomic level was categorized into quartiles and determined according to the poverty index of the member’s census enumeration area (small areas defined by the Israeli Bureau of Statistics for the 1995 national census data collection). The poverty index, ranging from 0 (lowest) to 20 (highest), is based on several parameters including household income, educational qualifications, crowding, material conditions, and car ownership (17). History of other comorbid conditions at index date, such as diabetes mellitus, cardiovascular disease, hypertension, or obesity, was identified on the basis of outpatient diagnoses. Information on health service use, such as data on hospitalizations in general hospitals or visits to outpatient clinics during the year before the index date, was collected from personal medical files.

Chi-square test for categorical variables and Kruskal-Wallis test for continuous variables were performed to determine significant differences in baseline characteristics among quintiles of PDC. To address the effect of statin type, we conducted sensitivity analyses of simvastatin users (n = 159,197). Cox’s proportional hazards (18) model with years of follow-up as the time scale was used to estimate hazard ratios (HRs) and 95% confidence intervals (CIs) and to identify variables significantly associated with cancer incidence. The full multivariable model included the following baseline values: age at baseline (in 1-year intervals), sex, marital status, socioeconomic level by quartile, presence of chronic comorbidity, use of health services, and efficacy of the initial statins therapy. To estimate the effects of smoking status we performed subanalysis for participants with smoking status in the models. Assumptions of proportional hazards were performed, and the ratio of hazards was the same across time. Data were analyzed with SPSS version 15 (SPSS Inc, Chicago, Illinois). The study was approved by the Assuta Health Systems Institutional Review Board.

## Results

During the follow-up period (952,202 person years [PY], a mean of 4.70 PY per patient), 9,256 patients (4.6%) died and 2,787 (1.4%) left MHS. The mean age of the total population was 57.3 years (Table 1). In general, patients in the highest PDC quintile were more likely to be older, men, or new immigrants, to belong to a higher socioeconomic level, and to have chronic diseases. Of the initial statin medications purchased by the 202,648 study participants, 159,197 (78.6%) were simvastatin.

**Table 1:** Demographic and Clinical Characteristics of Study Population, by Proportion of Follow-Up Days Covered (PDC) With Statins,^a^ Israel, 1998-2006

Characteristics at Index Date	Quintiles of PDC With Statins					

	1st (≤12%), n = 40,520	2nd (13%–39%), n = 40,539	3rd (40%–66%), n = 40,574	4th (67%–85%), n = 40,489	5th (≥86%), n = 40,526	Total, N = 202,648
Age, y, mean (SD)	53.6 (14.2)	56.0 (12.2)	57.65 (11.6)	58.67 (11.2)	60.7 (10.8)	57.3 (12.3)
Men	47.3	48.3	48.2	50.6	52.9	49.5
Marital status^b^						
Never married	14.2	11.3	10.4	10.0	9.8	11.1
Ever married	85.0	87.4	88.4	88.6	88.7	87.6
**Socioeconomic level, quartile^c^**						
Lowest	30.8	32.0	28.2	25.2	21.7	27.6
2nd	32.1	33.8	33.2	31.3	29.3	31.9
3rd	14.2	13.7	13.9	14.3	14.0	14.0
Highest	22.9	20.5	24.6	29.3	35.0	26.5
**Statin efficacy**						
Low	34.9	39.0	35.0	32.5	30.1	37.8
Moderate	58.8	53.5	57.7	59.5	64.1	59.1
High	6.3	7.5	7.3	8.0	5.8	3.0
**Statin type**						
Lipophilic	92.0	92.4	92.4	92.8	91.9	92.3
Hydrophilic	8.0	7.6	7.6	7.2	8.1	7.7
**Statin group**						
Simvastatin	79.5	81.6	77.9	78.3	75.4	78.6
Atoravastatin	7.1	6.8	9.8	9.4	10.1	8.7
Pravastatin	7.9	7.4	7.4	7.0	8.0	7.6
Other	5.5	4.2	4.9	5.3	3.5	5.1
**Comorbidities**						
Hypertension	47.7	61.1	65.8	66.9	73.9	63.2
Cardiovascular disease	6.3	8.8	11.9	14.6	23.1	12.9
Morbid obesity	15.6	18.7	17.8	17.2	16.2	17.1
Diabetes mellitus	16.3	26.9	29.8	30.6	34.2	27.5
**No. of hospitalizations in the year before first statin dispensation**						
None	88.8	88.1	88.0	87.4	84.5	87.4
1	8.2	8.3	8.5	8.7	10.2	8.8
2	1.9	2.3	2.3	2.6	3.4	2.5
≥3	1.1	1.3	1.2	1.3	1.8	1.3
**No. of visits to physician in the year before first statin dispensation**						
<8	27.2	24.1	21.2	19.7	17.1	21.9
8-17	36.9	37.4	36.3	35.7	32.3	35.7
18–28	19.5	21.3	22.9	24.0	25.7	22.7
≥29	15.6	16.8	19.8	20.2	24.6	19.3

^a^ Calculated by dividing the quantity of statin packs dispensed, from date of first statin dispensation, by the total follow-up time. Data are presented as percentages unless otherwise indicated.

^b^ Data were missing for 2,543 respondents.

^c^ Socioeconomic level quartiles were 0-11 (lowest quartile); 12-14 (2nd quartile); 15-16 (3rd quartile); 17-20 (highest quartile).

A total of 8,662 incident cancer cases were reported during the follow-up period (Table 2). The incidence density rate of overall cancer was 9.10 per 1,000 PY (9.66 per 1,000 PY among men and 8.54 per 1,000 PY among women). Only 0.1% of cancers occurred within the first year of follow-up, whereas a total of 78.7% of cancers occurred after 3 years of follow-up. The most frequent types of diagnosed tumors in women were breast cancer (1,368 cases) and in men, prostate cancer (1,311 cases). Colorectal cancer was the most frequent type of malignancy among both sexes (1,247 cases). Among nonsolid cancers, the most frequent lymphomas were non-Hodgkin lymphoma (approximately 90%), and most leukemia cases were lymphocytic leukemia (40%) and myeloid leukemia (26.5%).

**Table 2:** Adjusted Hazard Ratios (HRs) and 95% Confidence Intervals (CIs) for Site-Specific Cancers, by Proportion of Follow-Up Days Covered (PDC) With Statins,^a^ Israel, 1998-2006

Site	Cases, n	PDC Quintiles	Adjusted for Age and Sex		Fully Adjusted^b^	

			HR (95% CI)	*P* Value	HR (95% CI)	*P* Value
All-site	8,662	1	1 [Reference]		1 [Reference]	
		2	1.03 (0.96–1.10)	.38	0.86 (0.80–0.93)	.001
		3	0.93 (0.87–0.99)	.001	0.75 (0.70–0.81)	.001
		4	0.82 (0.77–0.86)	.001	0.73 (0.68–0.79)	.001
		5	0.81 (0.76–0.86)	.001	0.90 (0.84–0.96)	.002

Colorectal	1,247	1	1 [Reference]		1 [Reference]	
		2	0.92 (0.76–1.11)	.41	0.89 (0.74–1.08)	.26
		3	0.80 (0.66–0.96)	.02	0.78 (0.65–0.94)	.01
		4	0.73 (0.61–0.89)	.001	0.73 (0.60–0.87)	.001
		5	0.93 (0.78–1.10)	.41	0.93 (0.78–1.11)	.43

Breast	1,368	1	1 [Reference]		1 [Reference]	
		2	0.86 (0.73–1.02)	.09	0.84 (0.71–0.99)	.03
		3	0.76 (0.64–0.90)	.002	0.73 (0.61–0.87)	.001
		4	0.81 (0.68–0.96)	.02	0.77 (0.65–0.92)	.005
		5	1.10 (0.93–1.29)	.27	1.03 (0.87–1.22)	.10

Prostate	1,311	1	1 [Reference]		1 [Reference]	
		2	0.89 (0.73–1.07)	.22	0.81 (0.67–0.99)	.04
		3	0.76 (0.63–0.92)	.005	0.72 (0.59–0.87)	.001
		4	0.82 (0.68–0.99)	.04	0.75 (0.62–0.89)	.002
		5	0.98 (0.84–1.16)	.86	0.95 (0.81–1.13)	.57

Lung and bronchus	584	1	1 [Reference]		1 [Reference]	
		2	0.82 (0.62–1.08)	.17	0.81 (0.61–1.06)	.14
		3	0.86 (0.66–1.13)	.30	0.85 (0.65–1.12)	.24
		4	0.72 (0.54–0.96)	.02	0.71 (0.53–0.94)	.02
		5	1.01 (0.79–1.29)	.93	0.97 (0.76–1.25)	.86

Female genital organs^c^	427	1	1 [Reference]		1 [Reference]	
		2	1.01 (0.75–1.37)	.93	0.89 (0.66–1.22)	.50
		3	0.97 (0.72–1.32)	.88	0.85 (0.63–1.16)	.33
		4	0.74 (0.53–1.03)	.07	0.65 (0.46–0.91)	.01
		5	0.88 (0.69-1.13)	.44	0.78 (0.57–1.06)	.12

Hematopoietic^d^	681	1	1 [Reference]		1 [Reference]	
		2	0.88 (0.69–1.13)	.32	0.87 (0.68–1.12)	.29
		3	0.74 (0.58–0.94)	.02	0.72 (0.56–0.92)	.01
		4	0.69 (0.54–0.89)	.004	0.65 (0.51–0.85)	.001
		5	0.76 (0.61–0.96)	.02	0.69 (0.55–0.88)	.002

Leukemia	177	1	1 [Reference]		1 [Reference]	
		2	0.72 (0.45–1.14)	.16	0.72 (0.45–1.15)	.17
		3	0.58 (0.37–0.94)	.03	0.58 (0.36–0.91)	.03
		4	0.55 (0.34–0.89)	.02	0.54 (0.33–0.88)	.01
		5	0.63 (0.61–0.96)	.03	0.58 (0.37–0.91)	.02

Lymphoma	429	1	1 [Reference]		1 [Reference]^e^	
		2	0.91 (0.67–1.24)	.55	0.87 (0.64–1.20)	.42
		3	0.87 (0.64–1.18)	.37	0.82 (0.61–1.11)	.21
		4	0.78 (0.57–1.07)	.14	0.72 (0.52–0.99)	.05
		5	0.78 (0.58–1.04)	.10	0.69 (0.51–0.94)	.002

^a^ Calculated by dividing the quantity of statin packs dispensed, from date of first statin dispensation, by the total follow-up time. Data are presented as percentages unless otherwise indicated.

^b ^Adjusted for age, sex, marital status, area of residence, nationality, socioeconomic level, years of stay in Israel, obesity, diabetes mellitus, hypertension, cardiovascular disease, efficacy, hospitalizations and visits to physicians a year before first statin dispensation, as well as for asthma, and chronic obstructive pulmonary disease (for lung and bronchus cancers).

^c^ Including uterus, ovary, cervix, fallopian tube, vagina, and vulva.

^d^ Leukemia, lymphoma, and multiple myeloma.

^e^ *P* for trend < .002.

After adjusting for potential confounders and statin efficacy, an inverse association between persistence with statin therapy and cancer risk was observed for all-site and site-specific cancers (Table 2). In a multivariable model, the highest cancer risk was calculated among nonpersistent statin users (lowest PDC quintile). However, we found no indication for a dose-response association between persistence with statin therapy and colorectal, breast, prostate, and lung cancers. Similar results were obtained when analyses were limited to patients with 3 or more years of follow-up and 5 or more years of follow-up and in subanalysis including only participants with known smoking status (data not shown). The sensitivity analysis included all study participants with smoking status (n = 63,863) with a total of 2,999 incident cancer cases. Of the 63,863 patients, 51,057 (79.9%) were never smokers, 4,166 (6.5%) were past smokers, and 8,640 (13.5%) were current smokers.

In the multivariable model, increased PDC quintile was associated with a significant risk reduction of all-site cancers with *P* = .001 for linear trend with an HR of 0.80 (0.76–0.86) for 5th PDC quintile compared with nonpersistent statin users (data not shown). The fully adjusted HR for hematopoietic cancers was 0.69 (95% CI, 0.55–0.88) for the highest PDC quintile; for lymphoma the HR was 0.69 (95% CI, 0.51–0.94, *P* = .002 for linear trend), and for leukemia the HR was 0.58 (95% CI, 0.37–0.91) (Table 2).

When PDC with statins was analyzed as a continuous variable, an increase of 10% in PDC level was associated with an adjusted HR of 0.98 (95% CI, 0.97–0.99; *P* = .02). In stratified analyses, substantially lower risk of cancer was calculated for patients aged 50 or older and for patients treated with high-efficacy statins (Figure).

**Figure:**
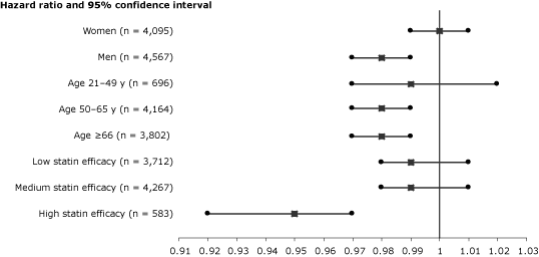
Proportional effects of persistence with statin therapy on reduction of risk for overall cancer per 10% of follow-up days covered with statins. Squares indicate adjusted hazard ratios for all covariates listed in Table 2. Horizontal lines indicate 95% confidence intervals. The 3 statin efficacy levels were created on the basis of expected amounts of low-density lipoprotein reduction from baseline.

**Table:** 

Cases of Cancer	Hazard Ratio (95% Confidence Interval)
**Women (n = 4,095)**	1 (0.99–1.01)
**Men (n = 4,567)**	0.98 (0.97–0.99)
**Age 21–49 y (n = 696)**	0.99 (0.97–1.02
**Age 50–65 y (n = 4,164)**	0.98 (0.97–0.99)
**Age ≥66 (n = 3,802)**	0.98 (0.97–0.99)
**Low statin efficacy (n = 3,712)**	0.99 (0.98–1.01)
**Medium statin efficacy (n = 4,267)**	0.99 (0.98–1.01)
**High statin efficacy (n = 583)**	0.95 (0.92–0.97)

Adjusted HR for all-site cancers and hematopoietic malignancies were stratified by sex (Table 3). Although the negative association between continuation with statin therapy and leukemia risk between the sexes was similar, significant differences were observed between men and women in relation to the risk of lymphoma. In men, increased PDC with statins was associated with lower risk of lymphoma, reaching approximately 40% lower incidence among adherent patients.

**Table 3:** Adjusted Hazard Ratios (HRs) and 95% Confidence Intervals (CIs) for All-Site Cancer and Hematopoietic Malignancies, by Proportion of Follow-Up Days Covered (PDC) With Statins,^a^ Stratified by Sex,^b^ Israel, 1998-2006

PDC Quintiles	Men		Women	

	HR (95% CI)	*P* Value	HR (95% CI)	*P* Value
**All-site cancers**	**4,567 cases**		**4,095 cases**	
1	1 [Reference]		1 [Reference]	
2	0.93 (0.84–1.03)	.15	0.86 (0.78–0.96)	.005
3	0.79 (0.72–0.87)	.001	0.78 (0.71–0.87)	.001
4	0.77 (0.69–0.85)	.001	0.78 (0.71–0.87)	.001
5	0.91 (0.83–0.99)	.04	1.01 (0.92–1.12)	.72

**Hematopoietic**	**378 cases**		**303 cases**	
1	1 [Reference]		1 [Reference]	
2	0.84 (0.61–1.16)	.29	0.88 (0.61–1.29)	.54
3	0.72 (0.53–0.99)	.50	0.75 (0.51–1.09)	.13
4	0.55 (0.39–0.76)	.001	0.85 (0.59–1.23)	.40
5	0.55 (0.40–0.76)	.001	0.97 (0.68–1.38)	.72

**Leukemia**	**113 cases**		**64 cases**	
1	1 [Reference]		1 [Reference]	
2	0.84 (0.48–1.47)	.55	0.47 (0.20–1.11)	.09
3	0.53 (0.29–0.96)	.04	0.64 (0.55–1.35)	.24
4	0.47 (0.26–0.86)	.01	0.67 (0.32–1.38)	.28
5	0.56 (0.32–0.96)	.04	0.56 (0.27–1.17)	.12

**Lymphoma**	**229 cases**		**200 cases**	
1	1 [Reference]		1 [Reference]	
2	0.78 (0.51–1.19)	.26	1.0 (0.63–1.60)	.98
3	0.83 (0.56–1.24)	.37	0.88 (0.55–1.41)	.60
4	0.60 (0.39–0.92)	.02	0.99 (0.63–1.55)	.95
5	0.53 (0.35–0.81)	.003	1.05 (0.67–1.65)	.82

^a^ Calculated by dividing the quantity of statin packs dispensed, from date of first statin dispensation, by the total follow-up time. Data are presented as percentages unless otherwise indicated.

^b^ Adjusted for all covariates listed in Table 2.

## Discussion

The results of our cohort study indicate that patients with longer continuation of statin therapy had a lower risk of cancer compared with nonpersistent users. Our results are similar to those of several smaller studies, including a nested case-control study (19) that demonstrated a lower cancer risk among statin users compared with bile acid–binding resin users and a cohort study of 12,251 statin users and 334,754 nonusers (20).

In a site-specific analysis, we found that persistent use of statins was associated with a significant decrement in the long-term risk of leukemia and lymphoma (mostly non-Hodgkin lymphoma). Early meta-analysis (21) of 14 studies (6 RCTs, 7 case-control studies, and 1 cohort study) published between 1996 and 2006 indicated an insignificant inverse association between statin use and the risk of hematologic malignancies. However, a more recent study from the Cancer Prevention Study II Nutrition Cohort (22) found that compared with nonusers, patients who used statins for more than 5 years had a significant 25% reduction for non-Hodgkin lymphoma. Also, an inverse association was reported among statin users for lymphoma in EPILYMPH (12), a multicenter case-control study. Moreover, in vivo and in vitro reports have provided evidence that statins inhibit the growth and promote the self-destruction of leukemia cells (23,24).

When our analyses were stratified by sex, the significant negative association between continuation with statins and lymphoma risk was limited to men only. The reduction of hematopoietic cancer risk by sex also has been reported in other studies of prescription medications (12,25), but few studies have addressed statins. An inverse relationship between risk of non-Hodgkin lymphoma and statins was reported in a study that compared 601 histologically confirmed incident cases of non-Hodgkin lymphoma and 717 population-based controls among Connecticut women (26). However, the association was limited only to women with short to moderate therapy periods. The potentially differential sex-specific effect of statins on non-Hodgkin lymphoma risks warrants further research.

Our study had several methodologic strengths, including its historical prospective design, a large and unselected study population, systematic data collection, and a long follow-up period. The threat of methodologic biases was further reduced by an individual evaluation of statin persistence based on dispensing information, which is the most feasible method of estimating medication use in large populations (27). The use of the ICR cancer reports also reduced the threat of outcome misclassification bias. To minimize the potential effect of indication bias, only new users of statins who had at least 1 dispensed prescription of statins during the study period were eligible for analysis. Finally, the exposure start date is equal to the day of study initiation to avoid immortal time bias (28).

However, some potential limitations should be discussed. Statin users are frequently under continuous surveillance of various specialists, and more screening tests could have led to surveillance bias. However, such bias is usually associated with earlier cancer detection and higher observed cancer risk and thus cannot explain the negative association between PDC with statins and cancer risk observed in our cohort. A healthy user bias is another potential bias. Persistent users may be more likely to have more aspects of a healthy lifestyle such as diet, exercise, and avoidance of risky behaviors (29). To avoid this bias, our study models were adjusted for visits to primary physicians during the year before the index date. Moreover, our study indicated that persistent use of statins is associated with reduced risk of hematopoietic neoplasms, for which screening tests are not available as they are for breast, colorectal, and prostate cancers.

Data on some variables that can be associated with statins and cancer, such as physical activity, diet, and family history of cancer, were missing in our analysis. However, none of these is an established risk factor for hematologic neoplasms. Smoking, a well-established risk factor for several types of cancer, is an additional confounder for which data were incomplete. Statins are more likely to be prescribed for cigarette smokers because of their higher risk of atherosclerotic cardiovascular disease. However, results from subanalyses for patients with valid smoking status were similar to overall analysis. The results of sensitivity analyses by follow-up duration suggest that the threat of methodologic biases such as misclassification of exposure were unlikely.

Our study showed that overall cancer risk decreased with increasing level of statin efficacy, but we found no significant differences between lipophilic and hydrophilic statins. Several studies (9,30) have also failed to demonstrate differences in cancer risk between statin types except for 2 cohort studies of lipophilic statin users, who had a reduced risk of breast cancer (8) and prostate cancer (31) incidence compared with nonusers.

In light of widespread statin consumption and the indications for long-term or lifelong use, the association between statin use and lower cancer risk may contribute to improved public health. For example, the incidence of leukemia in Israel is 32 per 100,000 for Jewish men aged 60 to 69 years (3). Using the calculated HR of 0.56 to calculate the absolute reduction in risk, we determined that statin therapy could prevent 14 cases per 100,000 Jewish men in this age group. The observed effect might be greater with the introduction of more efficacious statins in recent years. Additional controlled clinical trials are needed to investigate the potential anticancer benefit of statins, particularly in nonsolid tumors.

## Figures and Tables

**Table:** 

**1. The activity supported the learning objectives.**				
**Strongly Disagree**				**Strongly Agree**
1	2	3	4	5
**2. The material was organized clearly for learning to occur.**				
**Strongly Disagree**				**Strongly Agree**
1	2	3	4	5
**3. The content learned from this activity will impact my practice.**				
**Strongly Disagree**				**Strongly Agree**
1	2	3	4	5
**4. The activity was presented objectively and free of commercial bias.**				
**Strongly Disagree**				**Strongly Agree**
1	2	3	4	5

## Post-Test Information

To obtain credit, you should first read the journal article. After reading the article, you should be able to answer the following, related, multiple-choice questions. To complete the questions (with a minimum 70% passing score) and earn continuing medical education (CME) credit, please go to http://www.medscape.org/journal/pcd. Credit cannot be obtained for tests completed on paper, although you may use the worksheet below to keep a record of your answers. You must be a registered user on Medscape.org. If you are not registered on Medscape.org, please click on the "Register" link on the right hand side of the website to register. Only one answer is correct for each question. Once you successfully answer all post-test questions you will be able to view and/or print your certificate. For questions regarding the content of this activity, contact the accredited provider, CME@medscape.net. For technical assistance, contact CME@webmd.net. American Medical Association's Physician's Recognition Award (AMA PRA) credits are accepted in the US as evidence of participation in CME activities. For further information on this award, please refer to http://www.ama-assn.org/ama/pub/category/2922.html. The AMA has determined that physicians not licensed in the US who participate in this CME activity are eligible for **AMA PRA Category 1 Credits™**. Through agreements that the AMA has made with agencies in some countries, AMA PRA credit may be acceptable as evidence of participation in CME activities. If you are not licensed in the US, please complete the questions online, print the AMA PRA CME credit certificate and present it to your national medical association for review.

## Post-Test Questions

### Article Title: Statin Therapy and CA Risk

**CME Questions**

1. You are seeing a 57-year-old man with hyperlipidemia. You tell him that you want to prescribe a statin for him, and he has questions regarding potential benefits of statins apart from a reduced risk of cardiovascular disease. What should you consider regarding the background and mechanisms of statin therapy with regard to cancer prevention?
  - Cholesterol levels themselves are unrelated to cancer growth
  - Statins prevent the synthesis of mevalonic acid
  - No research to date has demonstrated that statin use may reduce the risk of cancer
  - Previous observational studies of statins and cancer incidence are of high methodological quality
2. As you discuss treatment with this patient, which of the following statements regarding the effect of statins on the risk of cancer in the current study is most accurate?
  - There was no significant association between statins and the risk of cancer
  - Any statin use was associated with a modest increase in the risk of cancer
  - There was a progressive effect of greater persistence of statin use in reducing the risk of cancer
  - Only patients who used statins at the highest doses experienced a lower risk of cancer
3. What can you tell this patient was the type of cancer most affected by the use of statins in the current study?
  - Hepatocellular cancer
  - Lung cancer
  - Hematopoietic cancer
  - Prostate cancer
4. Which of the following statements regarding subgroup analysis of the current study is pertinent for this patient?
  - The effect of statins on cancer risk was more pronounced among men vs women
  - The effect of statins on cancer risk was more pronounced among adults at age 30 to 39 years
  - The effect of statins on cancer risk was more pronounced among adults at age 40 to 49 years
  - The efficacy of statins did not change their effect on the incidence of cancer

**Evaluation**
